# Cattle NK Cell Heterogeneity and the Influence of MHC Class I

**DOI:** 10.4049/jimmunol.1500227

**Published:** 2015-07-27

**Authors:** Alasdair J. Allan, Nicholas D. Sanderson, Simon Gubbins, Shirley A. Ellis, John A. Hammond

**Affiliations:** The Pirbright Institute, Pirbright, Woking, Surrey GU24 0NF, United Kingdom

## Abstract

Primate and rodent NK cells form highly heterogeneous lymphocyte populations owing to the differential expression of germline-encoded receptors. Many of these receptors are polymorphic and recognize equally polymorphic determinants of MHC class I. This diversity can lead to individuals carrying NK cells with different specificities. Cattle have an unusually diverse repertoire of NK cell receptor genes predicted to encode receptors that recognize MHC class I. To begin to examine whether this genetic diversity leads to a diverse NK cell population, we isolated peripheral NK cells from cattle with different *MHC* homozygous genotypes. Cytokine stimulation differentially influenced the transcription of five receptors at the cell population level. Using dilution cultures, we found that a further seven receptors were differentially transcribed, including five predicted to recognize MHC class I. Moreover, there was a statistically significant reduction in killer cell lectin-like receptor mRNA expression between cultures with different CD2 phenotypes and from animals with different *MHC class I* haplotypes. This finding confirms that cattle NK cells are a heterogeneous population and reveals that the receptors creating this diversity are influenced by the MHC. The importance of this heterogeneity will become clear as we learn more about the role of NK cells in cattle disease resistance and vaccination.

## Introduction

Natural killer cells are a diverse lymphocyte population with fundamental roles in immunity, cancer, and reproduction (1). Extensive studies in humans and mice have shown that as part of the innate immune system, NK cells can recognize and kill transformed or infected cells, particularly those virally infected, and initiate subsequent immune responses through the release of cytokines. This cytokine release, in addition to direct interactions between NK cells and dendritic cells, also helps initiate the adaptive immune response mediated by B and T cells (2). In human reproduction, NK cells are also involved in forming the placenta through interaction with the extravillous trophoblast (1). These diverse NK cell functions are mediated by a plethora of activating and inhibitory cell surface receptors that recognize a diverse array of ligands. The balance of signals received from these receptors determines the activation status of an individual NK cell (3).

NK cells express a wide range of receptors and other cell surface markers, some of which are expressed on other lymphocytes, whereas others are restricted to NK cells and some T cell subsets. This is particularly true of the inhibitory receptors, which are often members of large polymorphic gene families, and a majority of their ligands are the polymorphic MHC class Ia molecules (4). The interaction between NK cell receptors and MHC class I is fundamental not only for the recognition and subsequent activation against target cells but also for functional education and receptor repertoire development (5–7). This highly polymorphic system creates differential specificity and avidity between receptor and ligand pairs within populations (8). Avidity is also influenced by the peptide presented by the MHC class I, which can significantly alter the extent of NK cell inhibition (9, 10). A further diversity-generating mechanism is the variegated expression of receptors between individual NK cells, creating a differentially responsive cell population (5, 11, 12). The overall outcome is immune diversity created by the variable expression of polymorphic germline-encoded receptors, which can contribute to differential susceptibility to viral diseases in humans and mice.

The extracellular domains of NK cell receptors that recognize MHC class I are either Ig-like or C-type lectin-like, and encoded within the leukocyte receptor complex (LRC) or NK complex (NKC), respectively. These gene complexes are located on different chromosomes in all mammals studied to date, and never on the same chromosome as the *MHC*. Simian primates, including humans, have evolved a diverse LRC-encoded killer cell Ig-like receptor (KIR) system. Rodents have convergently evolved an unrelated NKC-encoded Ly49 receptor system [encoded by the killer cell lectin-like receptor A gene (*KLRA*)] that recognizes MHC class I to control NK cell function (13). The lack of linkage between these codependent receptors and ligands can lead to individuals of a particular genotype containing receptors lacking strong ligands.

Studies in other species reveal that not all mammals have an expanded NK cell receptor gene family (14, 15). All the known gene expansions have occurred independently within receptor families predicted to bind MHC class I. In contrast to simian primates, the LRC of prosimians contains only one *KIR* gene, which is nonfunctional. However, alongside a nonfunctional *KLRA* gene, the NKC contains a large *KLRC/D* gene expansion (16). Horses are the only nonrodent species known to have expanded the *KLRA* genes (17). The most extensive expansion described to date is in cattle, the only species known to have significantly expanded two MHC class I receptor gene families, the *KIR* and *KLRC/D*, each encoded within a different gene complex (18–22).

Cattle classical *MHC class I* haplotypes—unlike those in humans and, to a large extent, mice—vary in the number of genes they contain (23). In humans the *HLA-A*, *B,* and *C* genes are highly polymorphic but present on almost all haplotypes. *MHC class I* diversity in cattle is generated by six relatively polymorphic classical genes, with between one and three present on any one haplotype. However, there is no evidence of one gene being more or less important for Ag presentation, although some genes appear dominant over others at restricting T cell responses to certain pathogens. This diversity presents significant challenges for the coevolution of germline-encoded MHC receptors that segregate independently. In cattle, this receptor ligand system has the potential to generate an enormous diversity of differentially responsive NK cells (23).

The influence of this diversity on cattle NK cell function is yet to be understood. Cattle NK cells are defined by their expression of NCR1, a member of the natural cytotoxicity receptor family (24). Early work revealed that CD2 expression differentiated phenotypic NK cell subsets; 80% of peripheral blood NK cells are CD2^high^ in a resting state, less activated by IL-2 stimulation and a poorer producer of IFN-γ than CD2^low^ cells, although both subsets are equally cytotoxic (25, 26). Very little is currently known about the expression of other cattle NK cell receptors (18, 19, 24, 27), particularly the KIR and KLRC/D receptors that are predicted to bind MHC class I. To begin to address this, we examined the transcription of a panel of 25 NK cell receptors and other NK cell–associated molecules between and within individual animals with different *MHC class I* genotypes.

## Materials and Methods

### Animals

Heparinized peripheral blood samples were obtained by venipuncture from 14 Holstein–Friesian cattle (*Bos taurus)* with no overt signs of disease at the time of sampling. These cattle were part of an established MHC-defined homozygous herd that was generated by backcrossing (28). All animal experiments were approved by The Pirbright Institute Ethics Committee and carried out in accordance with the U.K. Animal (Scientific Procedures) Act 1986.

### NK cell isolation

PBMCs were separated from whole blood by density gradient centrifugation (1.083 g/ml Histopaque; Sigma-Aldrich, Gillingham, U.K.). NK cells were positively selected from PBMCs by labeling with a mAb recognizing bovine NCR1 (CD335) (AbD Serotec, Kiddlington, U.K.) at 3 μg/ml and rat anti-mouse IgG1 MicroBeads (Miltenyi-Biotec, Bergisch Gladbach, Germany) at 2.5 μl/10^7^ cells, to purities >85%. These cells were then stimulated in RPMI 1640 plus 10 mM HEPES plus 50 μM 2-ME (Sigma-Aldrich) containing either recombinant bovine IL-2 (0.5 μg/ml), a mix of recombinant bovine IL-12 and recombinant human IL-18 (eBioscience, San Diego, CA) (both 0.1 μg/ml) or recombinant human IL-15 (0.1 μg/ml) (eBioscience) for up to 7 d.

### NK cell dilution cultures

To generate single-cell NK cell cultures, NK cells positively selected from PBMCs were taken after 24-h cytokine stimulation (either IL-2 or IL-15) for limiting dilution assays. Cells were diluted in 96-well plates to 10 cells per 100 μl, 1 cell per 100 μl, or 0.33 cell per 100 μl before the addition of 5 × 10^4^ allogeneic irradiated PBMC feeder cells and the same cytokine they were stimulated in overnight. Wells exhibiting growth after 14 d were expanded on 1 × 10^6^ fresh allogeneic feeder cells for an additional 14 d, before total cell counts were taken and a single NK cell origin and NCR1 expression were assessed by flow cytometry.

### Flow cytometry

NK cell and NK cell dilution culture subsets were identified by labeling with mAbs CD335-PE (NCR1; AbD Serotec), CC42-AF647 (CD2; conjugated using AF647, Life Technologies, Carlsbad, CA), and CC63-APC Cy5.5 (CD8; conjugated using APC Cy5.5, Innova Biosciences, Cambridge, U.K.). TRT1 (anti-turkey rhinotracheitis) (IgG1) and TRT6 (IgG2a) were used as isotype control mAbs. Cell populations were visualized using forward light scatter (FSC)–A and side light scatter–A to identify lymphocyte populations before removing cell doublets using FSC-A and FSC-H. Cell viability was confirmed using the nuclear stain DAPI. Quadrants and gates for NCR1^+^ and CD2 expression were determined by gating on Ig-matched isotype control Abs TRT1 (IgG1) and TRT6 (IgG2a), respectively (Supplemental Fig. 1). For NCR1 and CD2 profiling of ex vivo and in vitro stimulated MACS-isolated NK cells, ≥50,000 events within the live gate were counted. For NK cell limiting dilution cultures, ≥20,000 events were counted. Flow cytometry was performed using an LSRFortessa II (Beckton-Dickinson, Oxford, U.K.) and analyzed using FCS express v3 (De Novo software, Los Angeles, CA) or FlowJo v10.2 (TreeStar, Ashland, OR).

### RNA isolation, quality assessment, and cDNA synthesis

A total of 3 × 10^6^ cells from each NK cell population were resuspended in 1 ml TRIzol (Life Technologies), and RNA was extracted according to the manufacturer’s protocol. RNA quality and concentration were assessed using a NanoDrop spectrophotometer (Thermo Scientific, Wilmington, DE). cDNA was synthesized from 1 μg RNA using a SuperScript II Kit with oligo (dT_12–18_) primers (Life Technologies) according to the manufacturer’s guidelines.

### Primer generation and PCR

PCR primers for 25 NK cell receptor genes or NK-associated genes were adopted from previous studies (18, 19) or designed based on sequences deposited in GenBank (Table I). PCR conditions were optimized using cDNA and constructs containing the gene of interest. It was not possible to distinguish between some highly homologous genes and prevent primers amplifying more than one gene. These primers are denoted as KLRC1*2-7, KIR3DXL2/4, or KIR3DXL3/5/7, depending on the genes amplified by the primers (Table I). PCR using a cDNA template (100 ng) was carried out in a final volume of 25 μl containing 1× PCR buffer (20 mM Tris HCl, pH 8.4, and 50 mM KCl; Life Technologies), 2.5 mM MgCl_2_, 0.25 mM 2′-deoxynucleoside 5′-triphosphate, 1 μM each primer, and 1.25 U Taq polymerase (Life Technologies). The thermal cycling profile was as follows: 95°C for 1 min, 35 cycles of 95°C for 30 s, optimized primer-specific melting temperature for 30 s, 72°C for 30 s, followed by 72°C for 7 min. All reactions were run in a Veriti 96-well Thermal Cycler (Applied Biosystems, Foster City, CA). Specific amplification was confirmed by Sanger sequencing for each primer pair on at least two animals (Source Bioscience, Nottingham, U.K.).

### Statistical analysis

Statistical analysis was performed using Prism v6 (GraphPad Software, La Jolla, CA) and R v2.15.2 (http://www.r-project.org). Boolean analyses were performed using a custom python script (http://www.python.org) to sort NK cell receptor transcription profiles based on *MHC class I* genotype and the putative signaling function of the NK cell receptor gene. Python scripts are available on request.

## Results

### NK cells from MHC class I homozygous cattle have a conventional CD2 and CD8 expression profile

**FIGURE 1.**
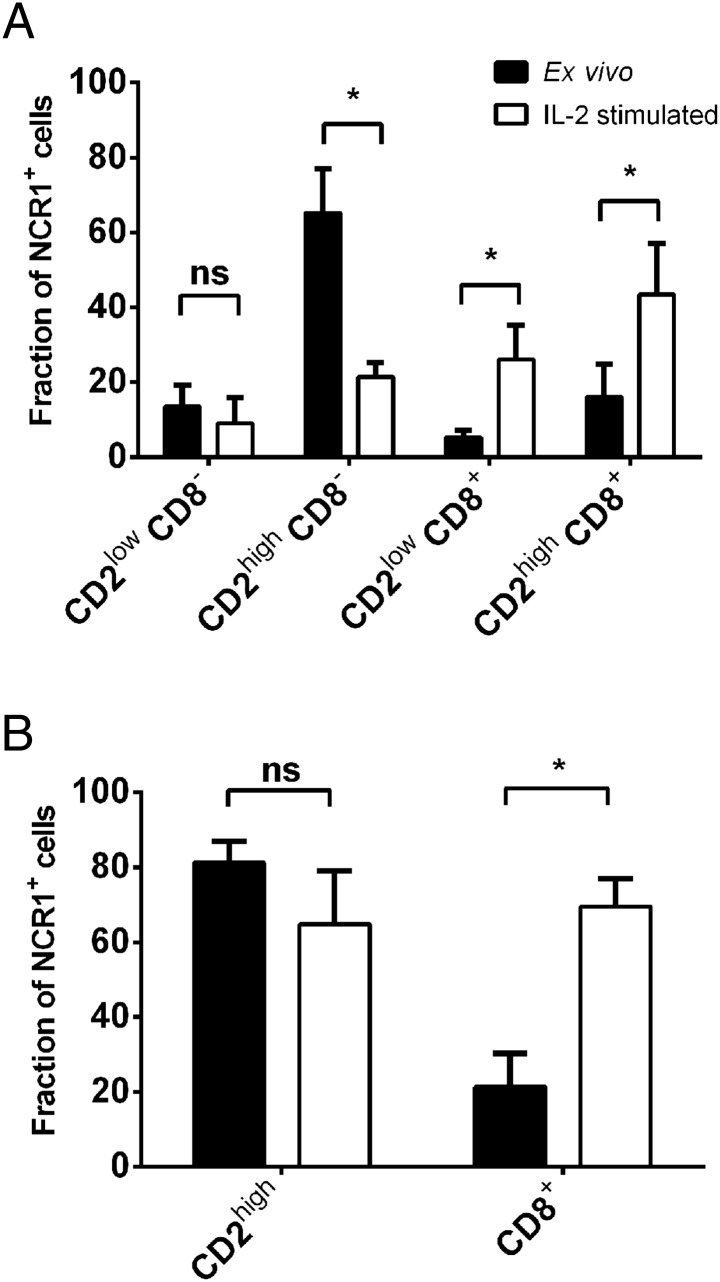
Ex vivo NK cells are predominantly CD2^high^, and CD8 expression increases upon stimulation with IL-2. PBMCs from 14 MHC class I–defined cattle were assessed for the expression of NCR1, CD2, and CD8 immediately ex vivo (unstimulated, filled bars) or after 7-d in vitro IL-2 stimulation (open bars). (**A**) The percentage of CD2^high^ and CD8^+^ cells within the NCR1^+^ population. Statistical significance was determined using a linear mixed model, with expression as the response variable and CD2/CD8 status and stimulated/unstimulated as fixed effects, plus an interaction between them and the animal as a random effect. (**B**) Overall change in the percentage of CD2^high^ and CD8^+^ within the NCR1^+^ population after cytokine stimulation. Statistical significance was determined using paired *t* tests. *p < 0.001.

As this study exploited a specially bred herd of *MHC* homozygous Holstein-Friesian cattle, it was important to establish that NK cells isolated from these animals were not substantively different from those used in previous studies (25, 26). Cattle NK cells are defined as NCR1^+^, with variable CD2 and CD8α expression (25, 26). We determined the cell surface expression of CD2 and CD8α on ex vivo and in vitro stimulated NCR1^+^ PBMCs from 14 *MHC* homozygous cattle. These animals represented three different haplotypes, four A18 containing only gene 6, four A14 containing a gene 1, 2 and 4 and six A31 containing a gene 1 and 2 but with different alleles to the A14. In all the animals, ∼80% of NCR1^+^ cells were CD2^high^ ex vivo and CD2 expression was reduced nonsignificantly (*p* = 0.27) after stimulation with IL-2 (Fig. 1). In contrast, CD8α expression increased in vitro under IL-2 when compared with ex vivo cells (*p* < 0.001) (Fig. 1), as has been shown with other cytokines (25). These NK phenotypes are consistent with previously published data showing that NK cells from these homozygous cattle have a broadly conventional phenotype and are responsive to cytokine stimulation.

### Receptor transcription in NK cells with divergent MHC class I genotypes are indistinguishable

To determine the receptor mRNA expression profile of ex vivo NK cells, the transcription of 25 NK cell receptor genes and other NK cell markers (Table I) were assessed immediately ex vivo and after cytokine stimulation in 14 cattle. Analysis by FACS confirmed that these cells were expressing NCR1 on the cell surface. For the purposes of this study, we divided these genes into three groups: the conventional NK cell molecules (CKM), the KLR, and the KIR. Although the majority of genes were transcribed regardless of *MHC class I* genotype or cytokine stimulation, some exceptions were noted.

**Table I.** NK cell receptor and associated genes studied and the primers used for PCR amplification

Gene(s)	Sense Primer (5′–3′)	Antisense Primer (5′–3′)	Accession/Reference No.
KIR3DXL2 KIR3DXL4	TCGGTGTCACTCCCGTTCTC	GTCTGCACGCAGTGATCCC	19
KIR3DXL3 KIR3DXL5 KIR3DXL7	GTCTCTCCCTGTGTTTTCCAGAAGCAG	ACTTTGGCGAGTGCAGGC	19
KIR2DL1	GTGGTTCCTCAAGGACAGCAT	CAGGGAGGACCCGTGGTG	19
KIR3DXL6	CAGACCGTGACTCTTTGG	GCGGGGAAAGAATGTGACC	19
KIR3DXL1	GTATGCCCCAGCTGACTTCTC	TGTACCACGTGCTRGAGTGTG	19
KIR3DXS1	GTATGCCCCAGCTGACTTCTC	AGCTGKCTGGAGTGGCCTT	19
NCR1	TTGTCCTTGGGCTGGGTC	GTAGTCAGAAGAGGTGTGC	AF422181.1
2B4	TGACCACCAAGATTTCACCTT	GCTGATTCCCAAAGCAGAAG	NM_001192350.1
CD16	GTCCACGTAGGCTGGCTATT	CTTCACGGTTGGCACTTTCT	AF132036.1
TIM-3	CAGAATGCNNATCTGCCCTGC	GGTTGGCCAAAGTGAYRAGGC	AB689695.1
KLRA*01	GCCAAAGTCACAATTCGTG	GCAATCAATTCTCTTCTCACAGATAC	AY075101
KLRA*02	GCCAAAGTCACGATTCATC	GCAATCAATTCTCTTCTCACAGATAC	GQ339253
KLRD1	ACCCTCTGCAGATCTTCAG	TATGCATCCAAAATCCTTCCTC	18
KLRD2	ACCCTCCACAGACCTCAGT	TCTTCATTCAATGCACCTCTG	18
KLRC1*1	GGTTCCATTTCAATAGCTCCA	TTCAAAAATAATACAATAGCAG	18
KLRC1*2-7	TAAAAGTTCCATTTCAATGACT	CCAGGATCCCAGCAATGAA	18
KLRC2*1	ACTCCAGGAAACAGCAAGTT	GTTATTCTGTTCCTGTATTAGAGC	18
KLRC2*2	GGAGACAGCAAATGAGAGATTC	TCGGGAGCTTTGTTACCAG	18
KLRK	CAGGATCCACCATGAGGAGGCTCTTATCCA	TCTGAGAACACGGCTCTCG	NM_001075139.1
NCR3	CCTAGCAATGCAGATATCACCG	GAAGAACTTGGGATCACTGGG	BC109614.1
DNAM-1	ACACCATGGCCTCTAACGAC	AATGTCCTCTCCTGCACCAC	XM_002697693.
IFNγ	TTCAAATTCCGGTGGATGAT	CCTGAAGCGCCAGGTATAAG	EF675629.1
CD2	TTCCCCACCAAAGGTGAAGAC	TCCTACACAAATGCCGATGA	AY841149.1
CD8α	CGCAGACTAGGTCGGTCTCT	GTTCCGGCGGTAGCAGAT	BC151259.1
CD8β	TGAGTGTGGTTGATGTTCTTCC	ACACCCAAGGACACCAGAAG	BC134439.1
CD25	GGCTCAAGTGCATACGTGAA	GGCCACTGCAATCTGGTACT	BC133546.1

**FIGURE 2.**
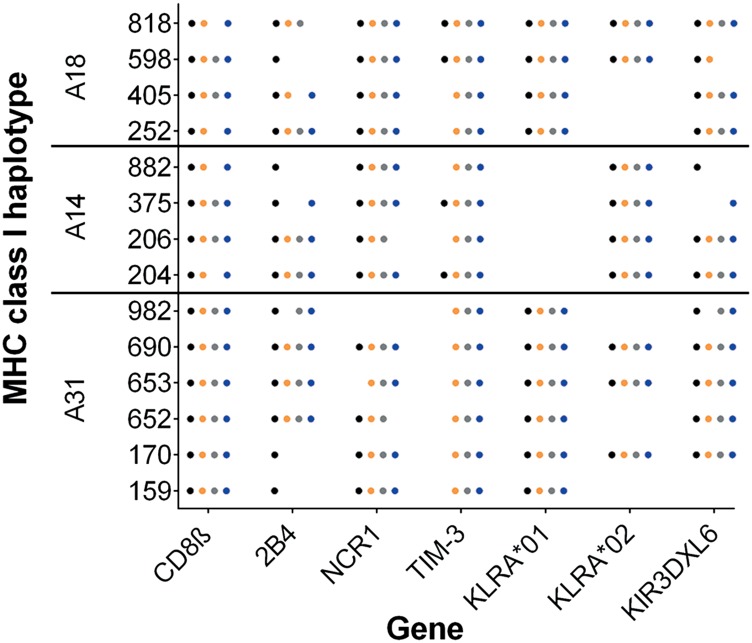
The variable transcription of NK cell receptor genes in ex vivo NK cells. The transcription of 25 NK cell receptor and associated genes was determined by PCR in NK cells from 14 MHC class I–defined animals (4 A18, 4 A14, and 6 A31). Genes that showed consistent variation are not shown. The dots represent transcription in immediately ex vivo NK cells (black), NK cells stimulated for 7 d in recombinant bovine IL-2 (orange), recombinant bovine IL-12, and recombinant human IL-18 (gray) or recombinant human IL-15 (blue).

Five genes showed variable mRNA expression patterns that were independent of *MHC class I* background (Fig. 2). NCR1 expression on CD3^−^ cells currently defines cattle NK cells, but we observed that NCR1 transcription was not ubiquitous, and in cells from animal 982 we were not able to detect NCR1 transcription following isolation based on NCR1 expression. The activating receptor gene *2B4* was always transcribed by ex vivo NK cells but was partially or completely lost in cytokine-stimulated NK cells from eight animals. Conversely, *TIM-3* was absent in the ex vivo populations from 10 of the 14 animals but was consistently transcribed by all animals after in vitro cytokine stimulation. CD8β was consistently transcribed by ex vivo and IL-2 and IL-15 in vitro stimulated NK cells, but in four animals, transcription was lost upon stimulation with IL-12/18. Only *KIR3DXL6* appeared to have variable transcription with no discernible pattern (Fig. 2). Although *KLRA* appears differentially transcribed, two distinct allele lineages are found in cattle. We have previously genotyped this group of animals for the presence of each *KLRA* lineage, and these NK population differences are due to genotype rather than differential regulation.

### Cattle NK cells are a diverse population

As in other species, differential receptor transcription is likely to create a heterogeneous NK population in cattle. To examine NK cell receptor transcription patterns at the single-cell level, we maintained a total of 67 NCR1^+^ short-lived limiting dilution cultures from two A14 and four A18 homozygous cattle, the two most divergent haplotypes in this study based on gene content. Cloning efficiency varied between animals and cytokine regimen, from 3% up to 25%, which is consistent with similar studies in mice (29). To confirm that these cells were of an NK origin, and not from the recently reported population of CD3^+^ NCR1^+^ T cells, we assessed each clone for transcription of the TCRβ-chain of the TCR, using previously published methods (30). TCRβ transcription was found in a single culture, 375_2H3, which was subsequently removed from further analysis. Flow cytometry of the remaining 66 dilution cultures confirmed that both NCR1^+^ CD2^high^ and NCR1^+^ CD2^low^ populations were present at a ratio of ∼75:25 (data not shown). This finding is consistent with our bulk culture data and the previously published ratio for ex vivo NK cells, confirming that both these major populations were at least partially represented (26).

**FIGURE 3.**
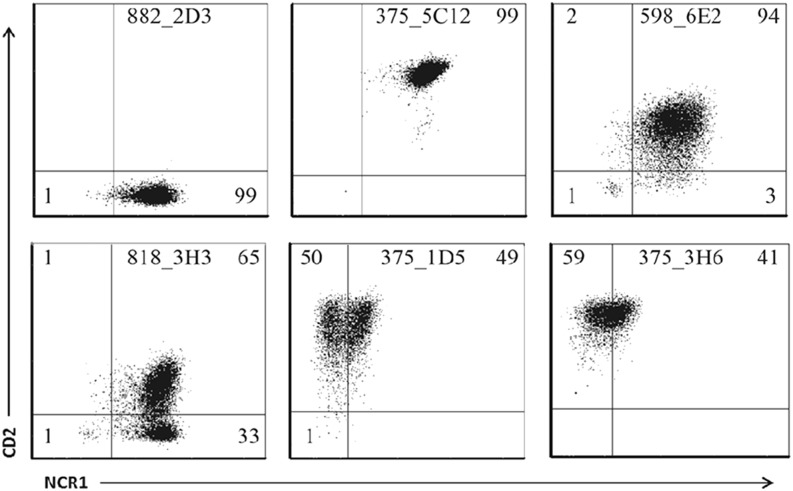
Individual dilution cultures have variable CD2 and NCR1 expression. Representative plots from six dilution cultures showing NCR1 and CD2 cell surface expression that was assessed by flow cytometry for each of the 66 limiting dilution cultures. Quadrants were set based on isotype matched control mAbs as described in *Materials and Methods*, with percentages shown in each quadrant.

Although a majority of the dilution cultures appeared to be from a single-cell origin, with a single phenotype determined by expression of NCR1 and CD2 (Fig. 3A–C), one had a mixed CD2 phenotype (Fig. 3D) and two had mixed NCR1 phenotypes (Fig. 3E, 3F). This finding could indicate a multiple-cell origin or the transient expression of NCR1 and CD2. As further limiting dilution was not possible, all 66 populations are referred to as dilution cultures rather than clones for the remainder of this article.

**FIGURE 4.**
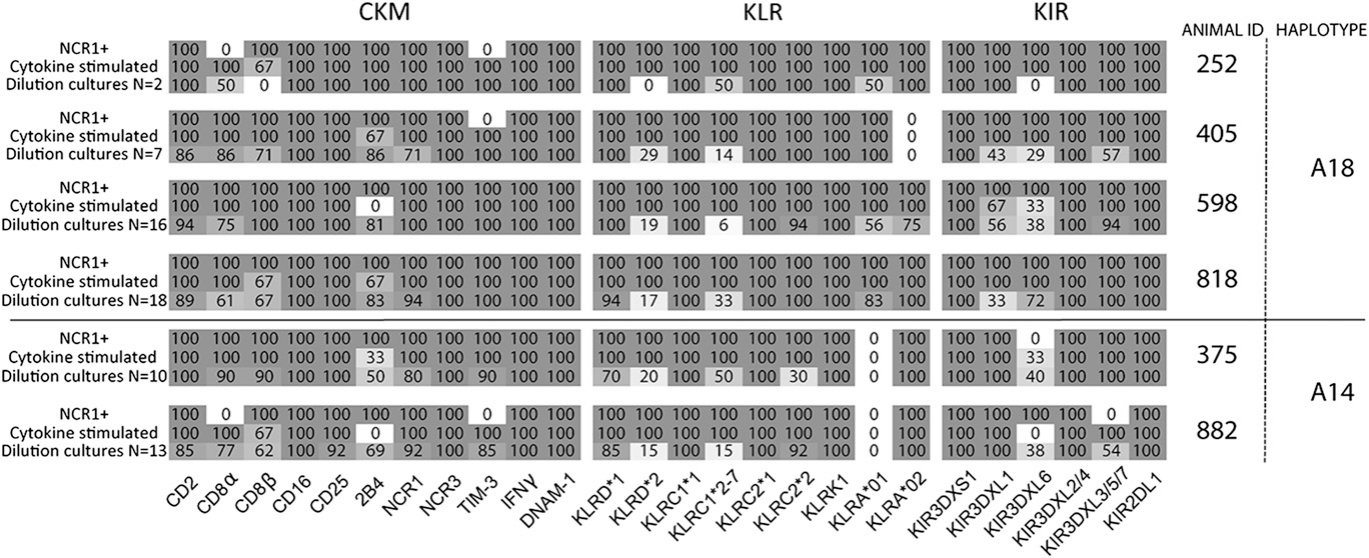
A summary of NK receptor transcription patterns from ex vivo, in vitro stimulated, and limiting dilution culture NK cells. Immediately ex vivo (NCR1^+^), NK cells that were stimulated in vitro in IL-2, IL-12, and IL-18 or IL-15 for 7 d (cytokine stimulated), and limiting dilution cultures (dilution cultures) from each animal are shown. Each square displays the percentage of transcription of that gene for that sample.

Transcription of the same 25 NK cell markers and receptors previously examined in ex vivo NK cells was determined (Fig. 4). A majority of the CKM genes were differentially transcribed; *CD2, CD8α, CD8β, CD25,* and *NCR1* were all variably present between dilution cultures from several individual animals. The variable pattern of *TIM-3* and *2B4* transcription was generally consistent between the dilution cultures and ex vivo NK cells from the same animals. Within the KLR group, mRNA for KLRC1*2-7, KLRC2*2, KLRD*1, and KLRD*2 was ubiquitously present at the population level but variably transcribed between the dilution cultures. Transcription of the *KLRA* alleles was consistent with the NK cell population data, with animals heterozygous for both allele lineages showing variable transcription patterns. As predicted from ex vivo NK cell populations, *KIR3DXL6* was variably transcribed between dilution cultures, as was *KIR3DXL1* and, to a more limited extent, the *KIR* group containing *KIR3DXL3/5/7*.

### NK cell receptor transcription is associated with CD2 expression and MHC class I genotype

**FIGURE 5.**
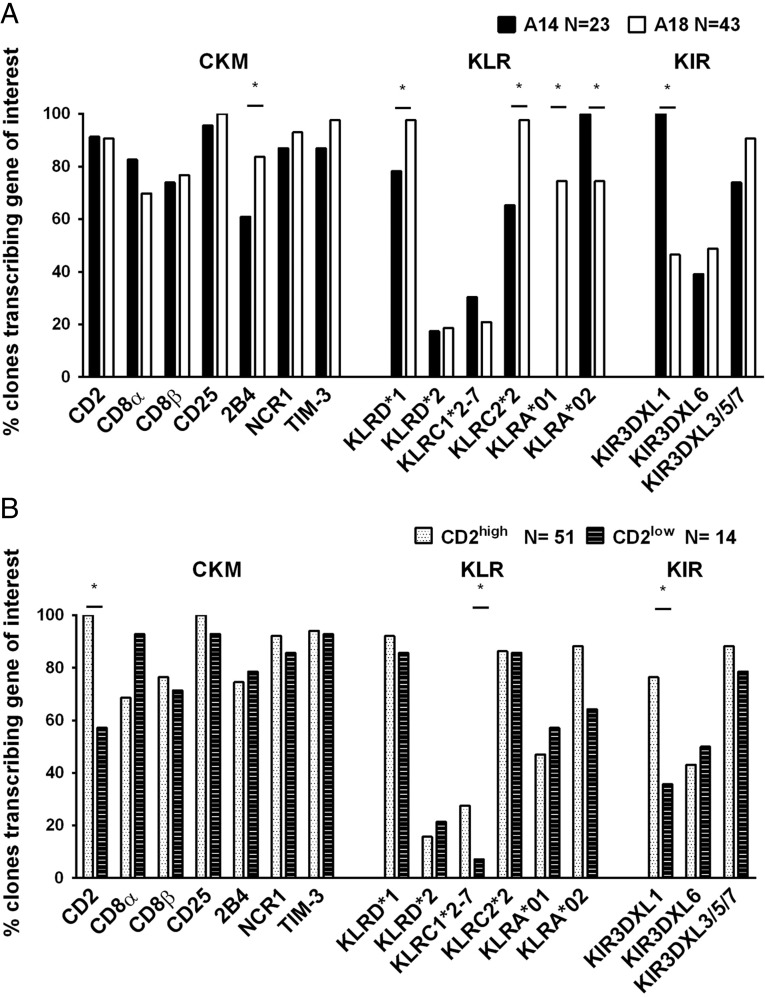
The frequency of dilution cultures transcribing NK cell receptors correlates with *MHC class I* genotype (**A**) and CD2 expression (**B**). Gene transcription is displayed as a normalized percentage of the total number of cultures within each population. Samples in which expression was mixed and genes were transcribed by all the dilution cultures were omitted. Statistical significance was determined using Fisher’s exact tests. *p < 0.05.

Having established NK cell receptor transcriptional variability within the dilution cultures, we examined whether there was any association between receptor transcription and NK cell phenotype or genotype. The expression of CD2 is an important differentiator of functional NK cell subsets in cattle (26). Therefore, we grouped the data from each dilution culture by CD2 expression and *MHC class I* genotype. Besides the significant association of *CD2* transcription with expression, KLRC1*2-7 and *KIR3DXL1* were significantly more likely to be present on the CD2^high^ NK cell subset regardless of *MHC class I* genotype (*p* < 0.05) (Fig. 5). The transcription of more inhibitory receptors correlates with the stronger inhibition of CD2^high^ NK cells compared with CD2^low^ (26).

The transcription of five receptor genes was also significantly correlated with *MHC class I* genotype (Fig. 5), using Fisher’s exact test. This correlation was independent of any animal-specific variation (Supplemental Fig. 2). The receptor *KIR3DXL1* and the KLRA*02 allele lineages were more likely to be transcribed in dilution cultures from A14 *MHC* haplotype animals. This observation is consistent with the lack of KLRA*01 in the A14 animals and very few CD2^low^ dilution cultures. However, KLRA*01, KLRD, and the activating receptors KLRC2*2 and *2B4* were all significantly more likely to be transcribed in the dilution cultures from the A18 animals. KLRD forms heterodimers with members of the KLRC family, and an increase in transcription of both KLRC2*2 and *KLRD* points to a potential functional interaction. Although it was not possible to measure a functional consequence of these differences using this system, we have identified genes that are not exclusively, but are preferentially, transcribed in NK cells from one *MHC class I* background over another, and between functionally different NK cell subsets.

**FIGURE 6.**
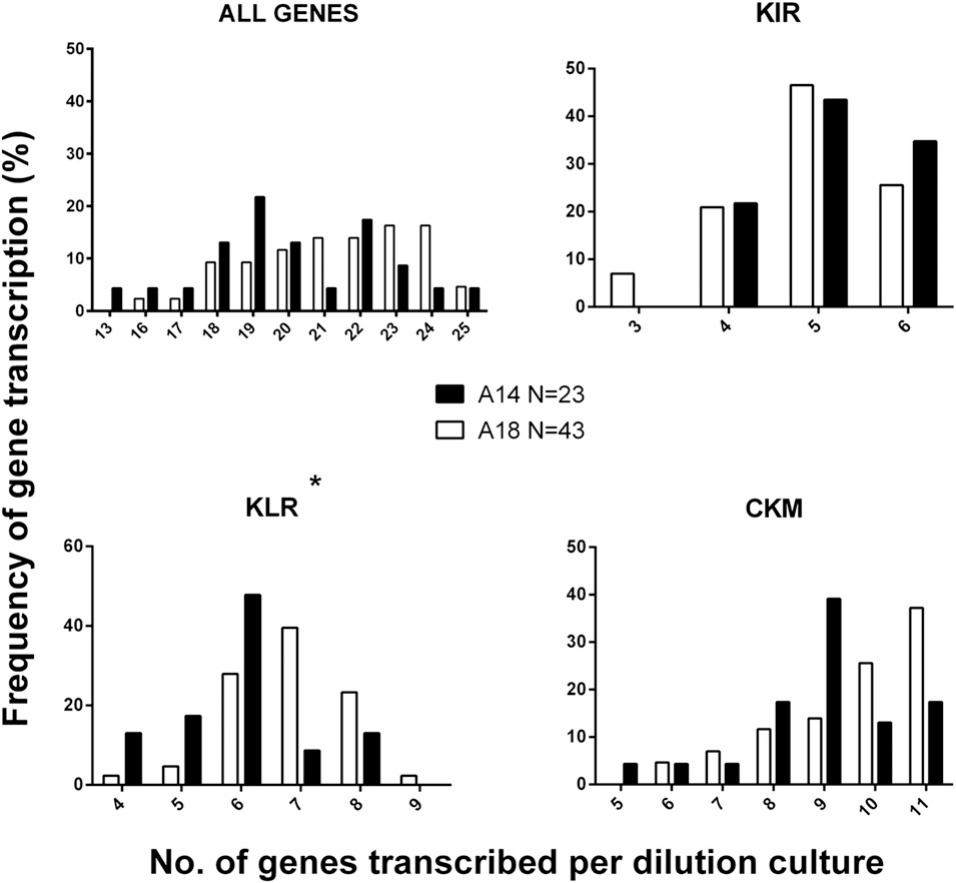
MHC class I haplotype influences the number of KLR receptors transcribed on individual dilution cultures. Dilution cultures were separated by *MHC class I* haplotype (A14, filled bars, and A18, open bars) and assessed for the total number of transcribed genes (*top left*) and also transcription of each NK receptor gene group: KIR, KLR, and CKM. The frequency has been calculated based on a normalized percentage of the dilution cultures with statistical significance between *MHC class I* haplotypes determined by a Fisher exact test. *p < 0.05.

As the transcription of individual receptors was influenced by *MHC class I* genotype, we compared the entire transcription profiles by *MHC class I* background. Of the 66 dilution cultures described in this study, we observed 52 different transcription phenotypes using our panel of 25 genes, with no single phenotype seen more than twice (data not shown). When separated by *MHC class I* background, the A14 dilution cultures transcribed fewer genes than the A18, which was due to significantly less KLR gene transcription (*p* < 0.05) and less transcription of the CKM genes, which approached significance (*p* = 0.12) (Fig. 6).

The *KLR* group includes genes that can encode inhibitory (KLRC1) or activating (KLRC2) receptors, whereas the majority of the CKM are activating. Therefore, we separated the transcription profiles based on both signaling potential and *MHC class I* background (genes that did not fit into either group were excluded from this analysis; Supplemental Table I). Although no statistical differences were found between the MHC groups, when analyzed together there was a clear dominance of two activating receptor transcription phenotypes: all the genes analyzed or all the genes except CD25 (Supplemental Fig. 3). This finding suggests that cattle NK cell functional diversity is primarily driven by variable expression of inhibitory receptors.

## Discussion

NK cell receptor genes predicted to recognize MHC class I ligands are highly diversified in cattle (23). We hypothesized that some of these receptors would be differentially transcribed to create a diverse NK cell population. Therefore, we determined the transcription of 25 NK cell receptors and other associated molecules from cattle with different *MHC class I* genotypes. A small number of genes were differentially transcribed after cytokine stimulation of entire ex vivo NK cell populations. Using limiting dilution cultures, we further identified mRNA from activating and inhibitory receptor genes, including members of the *KIR* and *KLRC/D* families, differentially expressed within and between animals. Comparing receptor transcription patterns revealed that the *KLRC/D* genes were the most variably present and, importantly, were preferentially transcribed by A18 *MHC class I* genotype NK cells compared with the A14. Overall, we have shown that cattle NK cells differentially transcribe several NK cell receptor genes and that *MHC* genotype can influence the frequency of receptor mRNA expression in the total NK population.

Haplotypic diversity in combination with allelic polymorphism creates extensive *MHC class I* diversity in cattle herds and breeds. Different combinations of haplotypes and almost all cattle will be heterozygous at the *MHC* and will inevitably vary in the genes that are expressed and therefore in the repertoire of antigenic peptides that can be presented. Although it is difficult to disentangle the effects of intensive breeding programs, evidence exists that certain haplotypes are more beneficial based on their penetrance in the population and the hierarchy of gene presence (31, 32). Our data suggest that this diversity will influence the receptor diversity of the circulating NK cell population. The transcription patterns of the paired receptors *KIR3DXL1* and *KIR3DXS1* effectively highlight the influence of MHC class I. *KIR3DXS1* is the only functional activating KIR receptor gene in cattle (18, 33) and was ubiquitously transcribed in all the dilution cultures, whereas *KIR3DXL1* was variably present in A18 NK cells. The significant presence of *KLR* receptor mRNA in more cultures from A18 animals, as well as suggesting these are functionally important receptors for MHC class I, indicates that MHC class I is involved in the development and function of cattle NK cells. Between individuals within a population there is likely to be a range of NK cell specificities and activation thresholds that will create subtly different responses to individual pathogens. This observation also adds to the body of genetic and comparative evidence all pointing to determinants of MHC class I being the ligands for the KIR, KLRA, and KLRC/D receptors in cattle, as they are in other species (4).

The interactions between NK cell receptors and MHC class I have been extensively described in humans and mice. Recently, this work has advanced the missing-self hypothesis from which the concepts of NK cell education and licensing have arisen (7). The activation threshold for an individual NK cell is determined by the strength of inhibition, which in turn is determined by the number and avidity of expressed self-inhibitory receptors (34–36). If cattle NK cells are educated by MHC class I in an analogous mechanism, our findings may point to an important effect of MHC class I on the development of NK cell receptor repertoires, which in turn creates diversity in the immune response.

The method used to generate NK cell dilution cultures in this study relied on cytokine stimulation, and it must be assumed that this method has altered the resting phenotype of these cells. The dominance of the activation receptor phenotype including all, or almost all, the genes is likely a result of this. However, it is of note that the proportion of CD2^high/low^ cultures was consistent with the ratio seen immediately ex vivo. Furthermore, although we could study only what is likely to be a fraction of the total number of NK cell phenotypes, it can be assumed that a majority of those we did isolate are among the most common, at least of those that proliferate in response to cytokine stimulation, and functionally relevant.

Owing to the very high sequence similarity between receptor cDNA sequences, it was not possible to discriminate between certain groups of genes by PCR. In addition, the PCR methods used were not quantitative and simply measured transcript presence or absence. As a consequence, the diversity described in this article is likely to be an underestimate in terms of qualitative and quantitative gene transcription. However, it is likely that receptor transcription and expression for most genes are correlated. A study assessing the genetic control of human KIR expression found strong correlations between expression of KIR3DL1, KIR2DL3, and KIR2DS4 and transcriptional activity (37). This may not be the case for all the genes or individual animals though, as expression levels can even vary between alleles, regardless of transcript abundance (38). This finding adds to the potential phenotypic diversity of the cattle NK cell population.

This study provides evidence that cattle NK cells constitute a heterogeneous population that differentially expresses several receptors, including those for MHC class I. As the frequency of NK cells transcribing individual receptors significantly correlated with the *MHC class I* genotype of the animal, individual animals are likely to differ in the frequency of NK cell phenotypes. As we learn more about the functional consequences of this diversity in cattle, this information should be considered when studying responses to pathogens and vaccination.

## Supplementary Material

Data Supplement
