# Supplementary material for: Cattle NK Cell Heterogeneity and the Influence of MHC Class I
Source: J Immunol. 2015 Jul 27;195(5):2199–206. doi: 10.4049/jimmunol.1500227 (PMC4543905; doi:10.4049/jimmunol.1500227)
Supplement: Data Supplement [file JI_1500227.zip › JI_1500227_Supplemental_Material_1.pdf]

**Supplemental Data:**

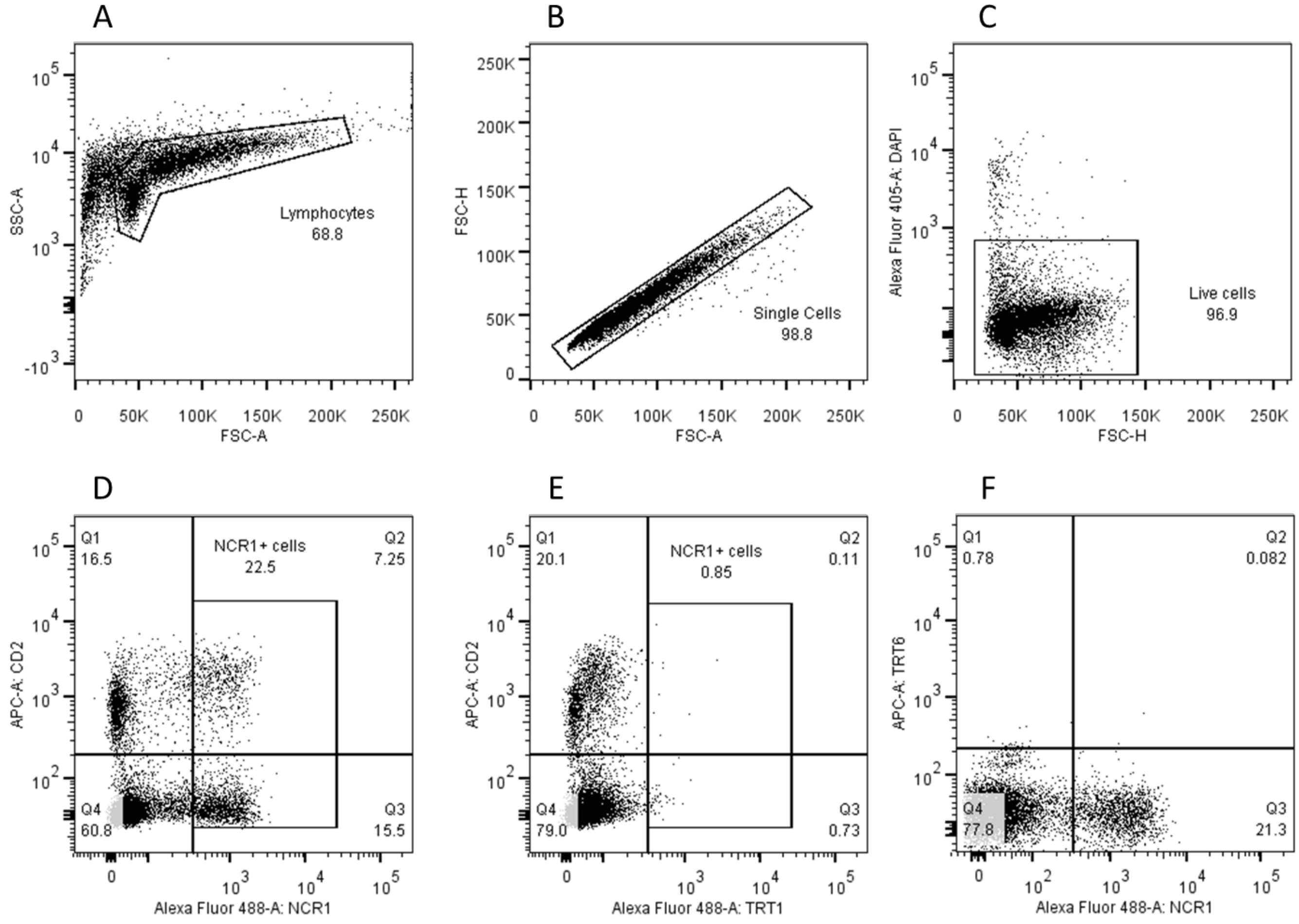


**Supplemental figure 1:** Flow cytometry gating strategies for NCR1 and CD2 expression on lymphocyte subsets. Cell populations were visualised using FSC-A and SSC-A to identify lymphocyte populations (A) before removing cell doublets using FSC-A and FSC-H (B). Cell viability was confirmed using the nuclear stain DAPI (C). Quadrants and gates for NCR1+ and CD2 expression (D) were determined by gating on Ig-matched isotype control antibodies TRT1 (E) and TRT6 (F) respectively. For NCR1 and CD2 profiling of *ex vivo* and *in vitro* stimulated MACS isolated NK cells (Figure 1) a minimum of 50,000 events within the live gate were counted. For NK cell limiting dilution cultures (Figure 3) a minimum of 20,000 events were counted. Representative plots from one of 14 animals are shown.

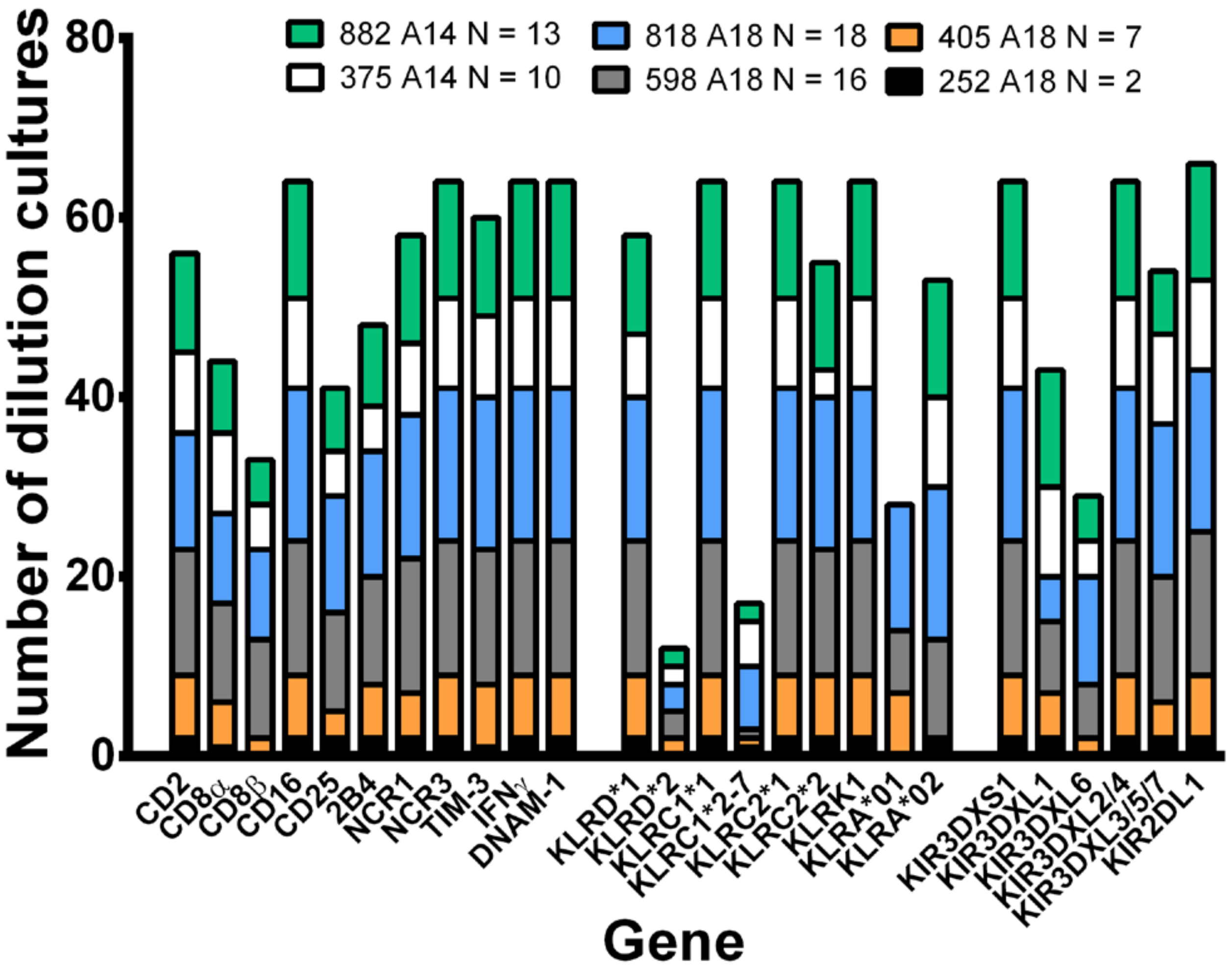


**Supplemental figure 2:** Receptor transcription patterns for each animal are broadly similar and not skewed by a small number of individuals. Each dilution culture has been colour coded by the animal of origin. The total number of clones from each animal is provided in the key.

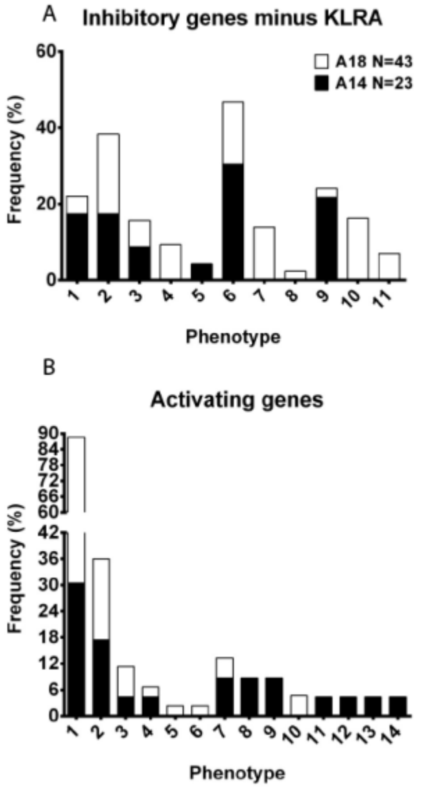


Inhibitory genes minus KLRA

| Phenotype | KLRC1*1 | KLRC1*2-7 | KIR3DXL1 | KIR3DXL6 | KIR3DXL2/4 | KIR3DXL3/5/7 | KIR2DL1 | Total genes transcribed |
|---|---|---|---|---|---|---|---|---|
| 1 | ✓ | ✓ | ✓ | ✓ | ✓ | ✓ | ✓ | 7 |
| 2 | ✓ | ✗ | ✓ | ✓ | ✓ | ✓ | ✓ | 6 |
| 3 | ✓ | ✓ | ✓ | ✗ | ✓ | ✓ | ✓ | 6 |
| 4 | ✓ | ✓ | ✗ | ✓ | ✓ | ✓ | ✓ | 6 |
| 5 | ✓ | ✓ | ✓ | ✓ | ✓ | ✗ | ✓ | 6 |
| 6 | ✓ | ✗ | ✓ | ✗ | ✓ | ✓ | ✓ | 5 |
| 7 | ✓ | ✗ | ✗ | ✓ | ✓ | ✓ | ✓ | 5 |
| 8 | ✓ | ✓ | ✗ | ✗ | ✓ | ✓ | ✓ | 5 |
| 9 | ✓ | ✗ | ✗ | ✗ | ✓ | ✓ | ✓ | 4 |
| 10 | ✓ | ✗ | ✓ | ✗ | ✓ | ✗ | ✓ | 4 |
| 11 | ✓ | ✗ | ✗ | ✗ | ✓ | ✗ | ✓ | 3 |

Activating genes

| Phenotype | CD16 | CD25 | 2B4 | NCR1 | NCR3 | TIM-3 | DNAM-1 | KLRC2*1 | KLRC2*2 | KLRK1 | KIR3DXS1 | Total genes transcribed |
|---|---|---|---|---|---|---|---|---|---|---|---|---|
| 1 | ✓ | ✓ | ✓ | ✓ | ✓ | ✓ | ✓ | ✓ | ✓ | ✓ | ✓ | 11 |
| 2 | ✓ | ✗ | ✓ | ✓ | ✓ | ✓ | ✓ | ✓ | ✓ | ✓ | ✓ | 10 |
| 3 | ✓ | ✓ | ✗ | ✓ | ✓ | ✓ | ✓ | ✓ | ✓ | ✓ | ✓ | 10 |
| 4 | ✓ | ✓ | ✓ | ✓ | ✓ | ✓ | ✓ | ✓ | ✗ | ✓ | ✓ | 10 |
| 5 | ✓ | ✓ | ✓ | ✗ | ✓ | ✓ | ✓ | ✓ | ✓ | ✓ | ✓ | 10 |
| 6 | ✓ | ✓ | ✓ | ✓ | ✓ | ✗ | ✓ | ✓ | ✓ | ✓ | ✓ | 10 |
| 7 | ✓ | ✗ | ✗ | ✓ | ✓ | ✓ | ✓ | ✓ | ✓ | ✓ | ✓ | 9 |
| 8 | ✓ | ✗ | ✓ | ✓ | ✓ | ✓ | ✓ | ✓ | ✗ | ✓ | ✓ | 9 |
| 9 | ✓ | ✗ | ✗ | ✓ | ✓ | ✓ | ✓ | ✓ | ✗ | ✓ | ✓ | 8 |
| 10 | ✓ | ✓ | ✗ | ✗ | ✓ | ✓ | ✓ | ✓ | ✗ | ✓ | ✓ | 8 |
| 11 | ✓ | ✗ | ✗ | ✗ | ✓ | ✓ | ✓ | ✓ | ✓ | ✓ | ✓ | 8 |
| 12 | ✓ | ✗ | ✗ | ✓ | ✓ | ✗ | ✓ | ✓ | ✓ | ✓ | ✓ | 8 |
| 13 | ✓ | ✓ | ✗ | ✓ | ✓ | ✗ | ✓ | ✓ | ✗ | ✓ | ✓ | 8 |
| 14 | ✓ | ✗ | ✗ | ✗ | ✓ | ✗ | ✓ | ✓ | ✗ | ✓ | ✓ | 6 |

**Supplemental figure 3:** The variable transcription of activating and inhibitory NK receptor genes in dilution cultures. Transcription of 25 NK receptor and associated genes were separated into three groups depending on the predicted signalling potential of the gene (supplemental table I). Individual phenotypes were then ascribed an arbitrary number and the phenotype with the highest number of genes transcribed listed as phenotype 1 and subsequent phenotypes are described in descending order of genes transcribed. Genes with inhibitory (A) and activating potential (B) are displayed. Gene transcription is displayed as a percentage of the total number of dilution cultures with MHC class I haplotype defined (A14 filled bars and A18 open bars). Individual NK receptor phenotypes and the total number of transcribed genes are listed in the tables on the right hand side of the figure.

## NK receptor gene signal classification

| Signal provided by gene | | |
| --- | --- | --- |
| **Activating** | **Inhibitory** | **Excluded** |
| CD16 | KLRC1*1 | CD2 |
| CD25 | KLRC1*2-7 | CD8α |
| 2B4 | KLRA*01 | CD8β |
| NCR1 | KLRA*02 | IFNγ |
| NCR3 | KIR3DXL1 | KLRD*1 |
| TIM-3 | KIR3DXL6 | KLRD*2 |
| DNAM-1 | KIR3DXL2/4 | |
| KLRC2*1 | KIR3DXL3/5/7 | |
| KLRC2*2 | KIR2DL1 | |
| KLRK | | |
| KIR3DXS1 | | |

**Supplemental table I:** The signalling classification of the 25 NK cell receptors used in this study.
